# Vaccination Attitudes in the Adult Population of Kazakhstan: A Nationally Representative Cross-Sectional Study

**DOI:** 10.3390/vaccines14040353

**Published:** 2026-04-16

**Authors:** Yerlan Ismoldayev, Anel Ibrayeva, Asset Izdenov, Sergey Lee, Altynay Sadykova, Bolat Sadykov, Shynar Tanabayeva, Ildar Fakhradiyev

**Affiliations:** 1 Department of Medicine, S.D. Asfendiyarov Kazakh National Medical University, Almaty 050000, Kazakhstan; 2 Department of Science, Ministry of Health of the Republic of Kazakhstan, Astana 010000, Kazakhstan; 3 College of Medicine, Korea University, Seoul 02841, Republic of Korea

**Keywords:** vaccine hesitancy, vaccination attitudes, Vaccination Attitudes Examination (VAX) scale, Kazakhstan, cross-sectional study, nationally representative survey

## Abstract

**Background/Objectives:** Vaccine hesitancy remains a significant public health challenge worldwide, yet nationally representative data from Central Asia are scarce. Evidence on the multidimensional structure of vaccination attitudes and their social patterning in Kazakhstan is limited. The study aimed to assess the distribution of anti-vaccination attitudes among adults in Kazakhstan and to examine their associations with socio-demographic, behavioural, clinical, and territorial characteristics. **Methods:** We conducted a cross-sectional, nationally representative survey of adults aged 18–69 years across all 17 regions of Kazakhstan between May and October 2025 (n = 6712). A multistage, stratified cluster sampling design was applied, and analyses incorporated sampling weights and design-based corrections. Vaccination attitudes were measured using the 12-item Vaccination Attitudes Examination (VAX) scale, comprising four subscales: mistrust of vaccine benefit, worries about unforeseen future effects, concerns about commercial profiteering, and preference for natural immunity. Internal consistency and confirmatory factor analysis were performed. Design-adjusted linear regression models were used to identify factors independently associated with each subscale and the overall VAX score. **Results:** The weighted mean overall VAX score was 3.70 (95% CI 3.67–3.73) on a 1–6 scale. The highest scores were observed for worries about unforeseen future effects (4.12; 95% CI 4.10–4.14), followed by preference for natural immunity (3.93; 95% CI 3.87–3.98), concerns about commercial profiteering (3.49; 95% CI 3.45–3.52), and mistrust of vaccine benefit (3.27; 95% CI 3.23–3.31). Internal consistency was high for the overall scale (Cronbach’s α = 0.861), and the four-factor structure demonstrated acceptable fit (CFI = 0.965; TLI = 0.952; RMSEA = 0.071). In multivariable design-adjusted models, age showed a generally consistent gradient, with lower scores in younger groups and the clearest differences observed among the youngest respondents. Married/cohabiting respondents had lower adjusted scores than single respondents across all subscales and for the overall VAX score. Men had lower adjusted worries scores than women, but sex was not independently associated with the overall VAX score. Diabetes was associated with higher adjusted mistrust, concerns about commercial profiteering, and overall VAX score, but not with worries or preference for natural immunity. Territorial differences were domain-specific: urban residence was associated with lower mistrust and higher worries, while macro-region was significant at the factor level only for worries. **Conclusions:** Anti-vaccination attitudes in Kazakhstan exhibit a multidimensional structure and clear socio-demographic patterning. Concerns about long-term safety were the most prominent attitudinal domain, whereas mistrust of vaccine benefit was comparatively less pronounced. Territorial differences were domain-specific rather than uniform, supporting the need for targeted communication strategies tailored to specific attitudinal domains and population subgroups.

## 1. Introduction

Vaccination remains one of the most important achievements of medicine, preventing an estimated 3.5 to 5 million deaths worldwide each year [1]. At the same time, vaccine hesitancy remains pronounced in contemporary populations: people delay or refuse vaccination even when vaccines are available [2]. The World Health Organization (WHO) included this behavior among the top ten threats to global health in 2019 [3]. Negative attitudes toward vaccination carry real risks: refusal of vaccination increases both the individual risk of severe disease and the societal burden of infectious illnesses. In recent years, researchers have increasingly sought to identify factors associated with anti-vaccination attitudes [4]. Socio-cultural characteristics such as age, sex, and education have been associated with attitudes toward vaccination, yet international studies have produced inconsistent findings. In some studies, women demonstrated higher levels of doubt and refusal than men, whereas in others the opposite pattern was observed [5,6,7]. Similarly, evidence on age remains mixed: some studies have found younger people to be more hesitant [8], while others have reported greater scepticism among older age groups [9,10]. These discrepancies suggest that vaccination attitudes change over time, differ across countries, and depend on the type of vaccine being considered [11]. In other words, global patterns of vaccine hesitancy are heterogeneous, underscoring the need for context-specific data. In addition, beyond demographic characteristics, vaccination behavior is also shaped by psychological factors, as personal traits and beliefs may directly influence attitudes toward immunization [12].

In the context of limited regional evidence on vaccine hesitancy, the local setting becomes especially important. For Central Asian countries, including Kazakhstan, data on public attitudes toward vaccination remain extremely limited [11,13]. In Kazakhstan, the available evidence has mainly addressed parental attitudes toward childhood vaccines and COVID-19-related hesitancy rather than broader anti-vaccination attitudes in the adult population. Therefore, nationally representative evidence on general vaccination attitudes among adults remains scarce.

Kazakhstan, the largest economy in the region and an upper-middle-income country, has a population of more than 20 million and is administratively divided into 17 regions and 3 cities of republican significance (Astana, Almaty, and Shymkent) [14]. At the same time, most of the population lives in urban areas. Recent immunization campaigns, both routine and COVID-19-related, have revealed pockets of concern and refusal. For example, Kazakhstan has experienced a resurgence of measles associated with growing vaccine hesitancy in the population [15]. In recent years, the number of recorded vaccination refusals increased markedly, reaching record levels of 42,282 cases in 2021 and 44,180 cases in 2022 [16]. Nevertheless, no comprehensive scientific studies assessing attitudes toward vaccination among the country’s adult population have yet been published. Understanding which population groups are characterized by higher levels of anti-vaccination attitudes is essential for more targeted public health planning.

The aim of this study was to assess anti-vaccination attitudes among adults in Kazakhstan and to examine their associations with socio-demographic, behavioural, clinical, and territorial characteristics. We also evaluated the internal consistency and factorial validity of the VAX scale in a nationally representative sample. We hypothesised that anti-vaccination attitudes would vary systematically across population subgroups rather than being distributed uniformly across the adult population.

## 2. Materials and Methods

### 2.1. Study Design and Sample

We conducted a cross-sectional, nationally representative survey of adults in Kazakhstan from May 2025 to October 2025. The final achieved sample included 6720 individuals aged 18–69 years selected using a multistage, stratified cluster design across all 17 administrative units of Kazakhstan, including the cities of Almaty, Astana, and Shymkent. Eight records with reported age > 69 years (outside the WHO STEPS target range) were excluded, yielding an analytic sample of 6712 participants. Survey weights were applied, and the complex sampling design (stratification and clustering) was accounted for to produce nationally representative estimates.

### 2.2. Sampling

A multistage cluster sampling design with stratification by sex (male, female) and age group (18–24, 25–34, 35–44, 45–54, ≥55 years) was used. The sample size was calculated using the World Health Organization STEPS sample size calculator (Excel-based tool) with the following parameters: 95% confidence level (Z = 1.96), assumed prevalence of 0.5, margin of error of 0.05, design effect of 1.5, and an anticipated response rate of 70%. The required minimum sample size was estimated at n = 6585. To ensure adequate regional representation and to compensate for non-response, the target sample size was increased to 6720. This 70% value also corresponded to the actual response rate achieved during the survey, and possible non-response was addressed within the sampling design, including through the use of reserve households.

**Figure 1:**
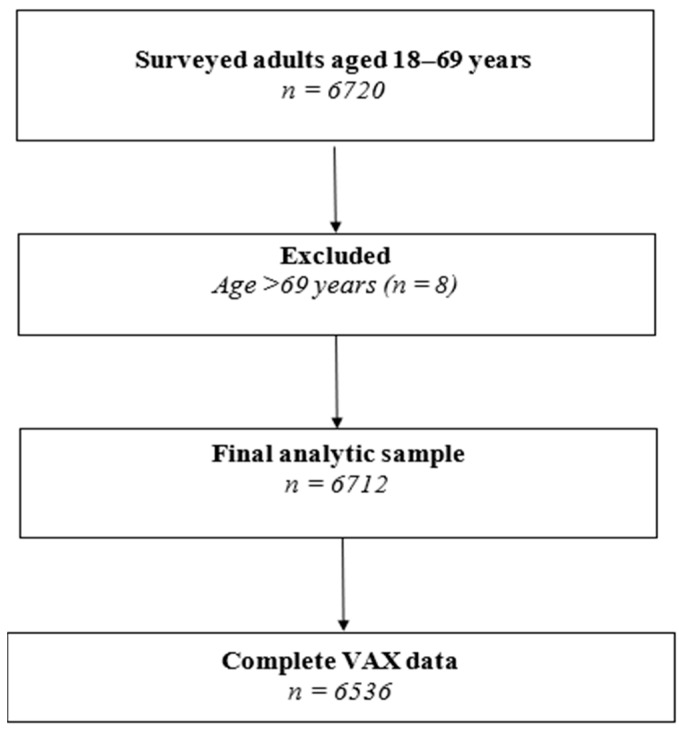
Flowchart of participant inclusion in the analytic sample.

Sampling was implemented in three stages. At the first stage, primary sampling units (PSUs), defined as districts and major urban centers, were selected with probability proportional to size using official population data from the Bureau of National Statistics of the Republic of Kazakhstan. At the second stage, primary health care (PHC) facilities within each selected PSU were chosen as secondary sampling units, proportionally to the size of the registered population, based on the national PHC registry. At the third stage, households within each selected PHC catchment area were randomly selected using the Randhold.xls tool, and one eligible adult per household was chosen using the Kish method. The planned number of households per PHC was approximately 28 (Figure 1).

### 2.3. Data Collection

Before the survey, data collection teams received training on interview techniques and physical/biochemical measurements. Interviewers explained the study’s goals to each household and obtained informed consent. Face-to-face interviews were conducted, and physical/biochemical measurements were taken on the same day.

### 2.4. Data Variables

The study followed the standardized WHO STEPwise approach [17]. The questionnaire was administered in Russian or Kazakh, according to participants’ preferences. In both cases, items were adapted from the original English WHO STEPS instrument using a standard forward–backward translation procedure. First, two bilingual physicians independently translated the English questionnaire into Russian and Kazakh, respectively, and any discrepancies between the forward translations were reconciled into single Russian and Kazakh versions. These preliminary versions were then back-translated into English by independent bilingual translators. The back-translations were compared with the original English questionnaire, and iterative revisions were undertaken until the research team agreed that both the Russian and Kazakh versions were conceptually equivalent to the source instrument.

In Step 1, trained interviewers collected socio-demographic data (age, sex, ethnicity, place of residence, education level, marital status, occupation) and information on behavioral risk factors (tobacco use, alcohol consumption). Steps 2–3 included physical measurements and venous blood sampling.

Region of residence was originally recorded according to all administrative units of Kazakhstan, including regions and cities of republican significance. For the main analysis, this variable was recoded into broader territorial categories to improve interpretability and ensure more stable estimates in design-based regression models. Specifically, regions were grouped into five macro-regions (North, Central, East, South, and West), while Astana, Almaty, and Shymkent were retained as separate categories because of their distinct urban and administrative profiles.

Anthropometric, blood pressure, and biochemical measurements were obtained according to WHO STEPS standard operating procedures. BMI was calculated from measured height and weight and categorized as underweight (<18.5 kg/m^2^), normal weight (18.5–24.9 kg/m^2^), overweight (25.0–29.9 kg/m^2^), or obesity (≥30.0 kg/m^2^). The hypertension status was classified as follows: normotension, mean systolic blood pressure (SBP) < 120 mmHg and diastolic blood pressure (DBP) < 80 mmHg; prehypertension, mean SBP 120–139 mmHg and/or DBP 80–89 mmHg; hypertension, mean SBP ≥ 140 mmHg and/or DBP ≥ 90 mmHg and/or treatment with antihypertensive drugs within the previous 2 weeks. Biochemical assessment included HbA1c and serum lipids collected according to standard laboratory procedures. Diabetes status was defined as HbA1c ≥ 6.5%, use of diabetes medication within the previous 2 weeks, or current insulin use; participants not meeting these criteria were classified as having no diabetes.

Current smoking was defined as a “yes” response to current tobacco use; HED was defined as ≥60 g of pure alcohol on at least one occasion in the past 30 days (WHO definition).

### 2.5. Vaccination Attitudes (VAX)

To assess participants’ attitudes toward vaccination, the Vaccination Attitudes Examination (VAX) scale was used. The study employed the Russian adaptation of the scale validated by Syromyatnikova et al., which demonstrated satisfactory psychometric properties [18]. The Kazakh version of the questionnaire was prepared using a forward–backward translation procedure involving bilingual specialists, following the adaptation methodology described by the authors of the Russian version, with subsequent expert reconciliation of item wording.

The scale comprises 12 statements rated on a 6-point Likert scale, ranging from “strongly disagree” to “strongly agree” (range 1–6). The items cover four components of anti-vaccination attitudes: (1) mistrust of vaccine benefit, (2) worries about unforeseen future effects, (3) concerns about commercial profiteering by vaccine manufacturers, and (4) preference for natural immunity over vaccination [18]. Items V1–V3 were reverse-coded.

Summary scores were calculated as mean values across items on the original 1–6 scale. The overall VAX score was defined as the mean of all 12 items after reverse-coding V1–V3, while each of the four subscale scores was calculated as the mean of the corresponding three items. Higher VAX scores were interpreted as indicating more pronounced anti-vaccination attitudes. The VAX scale assesses general attitudes toward vaccination and is not limited to any specific vaccine or vaccine-preventable disease.

The adaptation of the scale into Kazakh and Russian was conducted in accordance with the recommendations of the International Test Commission (ITC) [19]. Translation and cultural adaptation were performed using a back-translation approach with two independent bilingual translators, after which the item wording was harmonized through expert review.

Internal consistency of the overall VAX scale and its four subscales was assessed using Cronbach’s alpha. A threshold of α ≥ 0.70 was considered acceptable; in addition, corrected item–total correlations and the “alpha if item deleted” statistic were examined.

### 2.6. Statistical Analysis

The analysis was conducted in IBM SPSS Statistics version 24.0 using the Complex Samples module to account for the multistage stratified cluster design and sampling weights. The complex sampling design incorporated sampling weights, stratification variables, and clustering at the PSU level; standard errors and 95% confidence intervals were estimated using Taylor series linearization.

Descriptive statistics are presented as unweighted absolute counts (n) and weighted estimates (percentages or means) with 95% confidence intervals. For bivariate comparisons, design-adjusted procedures were applied: differences in mean scores were assessed using design-corrected F tests, and distributions of categorical variables were compared using the Rao–Scott χ^2^ test. Statistical significance was defined as *p* < 0.05 using a two-sided criterion.

To identify factors associated with anti-vaccination attitudes, separate design-adjusted multivariable linear regression models were fitted for each VAX subscale and for the overall VAX_total score as continuous outcomes. All covariates were entered simultaneously to estimate mutually adjusted associations. Results are presented as unstandardized regression coefficients (β) with 95% confidence intervals and *p*-values; global factor tests (Wald F), coefficients of determination (R^2^), and the overall model Wald F statistic were also reported.

Covariate selection and ordering were informed by a prespecified conceptual framework (Figure S1), which clarified the assumed temporal and structural relationships between socio-demographic, behavioural, and clinical characteristics and vaccination attitudes. Age and sex were considered baseline factors; socioeconomic variables were conceptualized as structural determinants; behavioural and clinical variables were treated as health-related characteristics potentially located along intermediate pathways. Given the cross-sectional design, all reported coefficients represent adjusted associations rather than causal effects. Given the number of tested associations across multiple outcomes and predictors, interpretation focused on effect sizes and confidence intervals rather than isolated *p*-values.

The assumptions of linear regression were evaluated by examining residual distributions and assessing multicollinearity. Variance inflation factors (VIFs) were calculated in parallel linear models; no evidence of problematic multicollinearity was identified (all VIFs < 2.5). Residual diagnostics did not reveal substantial departures from linearity or meaningful influence of outliers. Design-based standard errors estimated via Taylor series linearization account for clustering and stratification and are robust to heteroskedasticity.

### 2.7. Handling of Missing Data

Complete responses to all 12 VAX items were available for 6536 participants (97.4%). Analyses of the total VAX score and its subscales were restricted to respondents with complete VAX data. Psychometric analyses and multivariable regression models were conducted using a complete-case approach; therefore, the analytic sample size varied slightly across models depending on covariate completeness, and the corresponding n is reported in the relevant tables. Because the overall proportion of missing data was low and no evidence suggested a systematic pattern likely to invalidate complete-case analysis, imputation was not performed.

## 3. Results

### 3.1. Demographic Characteristics

The analytical sample included 6712 participants aged 18–69 years (mean age 41.6 ± 14.0 years; median 40 years) (Table 1). Based on weighted estimates, women constituted 52.4% (95% CI 51.2–53.6) of the study population, while men accounted for 47.6% (95% CI 46.4–48.8). A majority of respondents resided in urban areas (63.2%, 95% CI 62.0–64.3). Participants of Turkic ethnicity represented 76.9% (95% CI 75.8–77.9) of the population, whereas those of Slavic ethnicity accounted for 17.7% (95% CI 16.8–18.7). Most respondents were married or cohabiting (62.2%, 95% CI 61.0–63.4). More than half of the population had completed higher education (58.2%, 95% CI 57.0–59.4), and employment in the private sector was the most common occupational category (53.0%, 95% CI 51.8–54.2). With respect to health-related characteristics, 59.3% of participants were classified as having overweight or obesity, and 23.3% (95% CI 22.3–24.3) reported current tobacco use.

The overall VAX scale showed high internal consistency (Cronbach’s α = 0.861), with subscale α values ranging from 0.723 to 0.926 (Table 2). Most items demonstrated acceptable corrected item–total correlations. Item V4 showed comparatively weaker performance than the other items in its subscale; however, the “worries about unforeseen future effects” subscale remained within the acceptable range of internal consistency overall (α = 0.723) (Table S1). Confirmatory factor analysis supported the four-factor structure, with acceptable fit in the overall sample (CFI = 0.965, TLI = 0.952, RMSEA = 0.071).

The mean overall VAX score was 3.70. Scores were highest for worries about unforeseen future effects (4.12) and preference for natural immunity (3.93), whereas mistrust of vaccine benefit showed the lowest mean level (3.27) (Table 2).

Confirmatory factor analysis of the four-factor VAX model, estimated using maximum likelihood on complete observations, showed acceptable fit in the total sample (CFI = 0.965, TLI = 0.952, RMSEA = 0.071).

Across bivariate comparisons, anti-vaccination attitudes showed clear domain-specific patterning (Table 3). The most consistent gradients were observed for age and occupation, with higher scores in older respondents and generally lower scores among students. Women had higher worries scores than men, while clinical characteristics such as hypertension and diabetes showed higher mean scores in several domains. Territorial differences were also evident: rural residents had higher mistrust, whereas urban residents had higher worries.

### 3.2. Multivariable Regression Analyses

**Figure 2:**
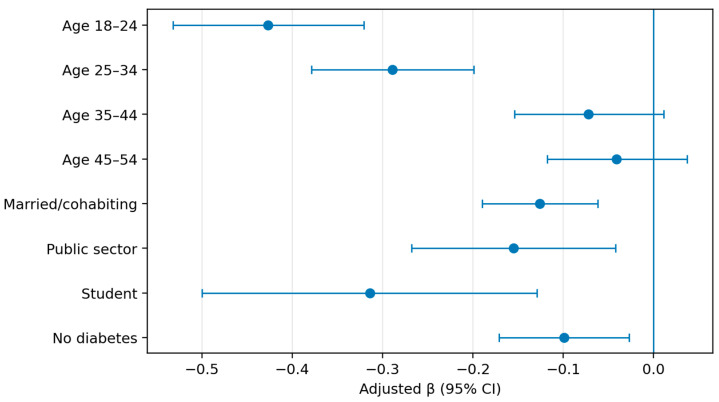
Adjusted regression coefficients (β) and 95% confidence intervals for factors associated with total VAX score (n = 6336).

**Figure 3:**
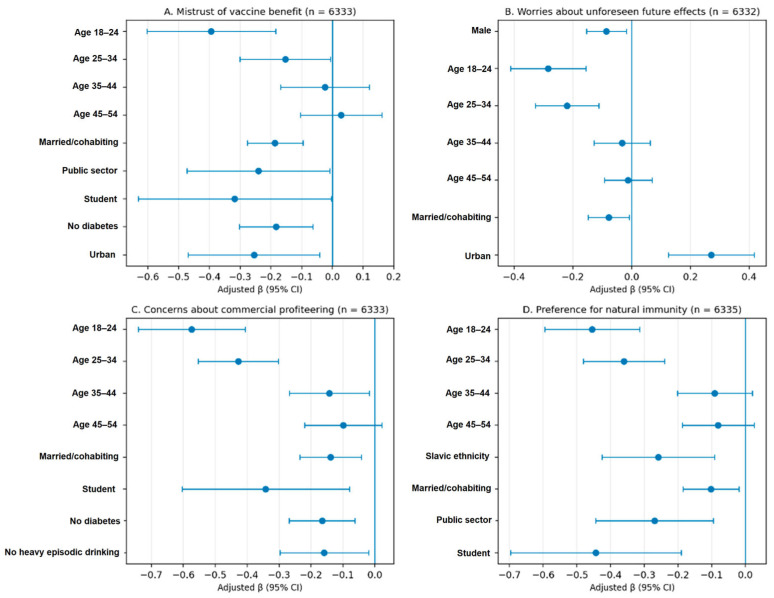
Adjusted regression coefficients (β) and 95% confidence intervals for key correlates of VAX subscales.

Multivariable regression models were estimated using complete-case analysis, with analytic sample sizes ranging from 6332 to 6336. Key adjusted associations are illustrated in Figure 2 and Figure 3, while full model coefficients are provided in Supplementary Tables S2–S6. Across all models, older age was the most consistent correlate of higher anti-vaccination attitudes, whereas married/cohabiting participants had lower scores across subscales and the total VAX score. Domain-specific patterns were also observed: men had lower scores only for worries about unforeseen future effects; urban residence was associated with lower mistrust but higher worries; diabetes was associated with higher mistrust, stronger concerns about commercial profiteering, and a higher total VAX score; and macro-region was significant only for worries at the global factor level.

## 4. Discussion

The present study is the first in Kazakhstan to provide a comprehensive assessment of anti-vaccination attitudes in a nationally representative sample of the adult population using the VAX scale and to examine their associations with a broad range of socio-demographic, clinical, and behavioural characteristics. These findings make it possible to move beyond fragmented observations of “vaccine hesitancy” toward an empirically grounded picture of how cognitive anti-vaccination attitudes are distributed across the population.

The weighted mean overall VAX score in the Kazakhstani sample was 3.70, with the highest values observed for worries about unforeseen future effects (4.12) and a relatively higher mean score for preference for natural immunity (3.93), whereas mistrust of vaccine benefit was more moderate (3.27). This configuration suggests not a radical rejection of vaccination, but rather an anxious and ambivalent attitudinal profile in which concerns about safety and the perceived “naturalness” of immunity play a central role.

A similar four-factor structure was described in the original study by Martin and Petrie [19]; however, the magnitude of these attitudes in the Kazakhstani population appeared higher. In US samples, the overall VAX score ranged from 2.77 to 3.20, and subscale values, particularly for mistrust and profiteering, were lower. Likewise, in the German population validation study, the overall score ranged from 2.80 to 2.89 [20], while among Italian nurses it was 2.93 ± 1.01 [21]. Although mean VAX scores in this study appear somewhat higher than some published estimates from European settings, direct cross-country comparisons should be interpreted cautiously because the studies differed in period, social context, and population composition.

The present findings are also consistent with evidence from large British panel studies in which worries about unforeseen future effects and mistrust of vaccines were statistically associated with uncertainty and refusal of vaccination [22]. However, in the British sample, the proportion of individuals with “high” subscale scores (5–6 points) was limited, whereas in Kazakhstan the mean values for the leading subscales were shifted toward the range of 3–4 and above, suggesting a broader distribution of ambivalent and critical attitudes within the population. Particularly noteworthy is the fact that the “preference for natural immunity” domain, which in some Western studies tends to show only moderate values, ranked second in mean score in the Kazakhstani sample. This may be related to culturally shaped beliefs about the “naturalness” of illness and immune response. Sex differences were limited and domain-specific rather than generalised. Women reported higher worries about unforeseen future effects, whereas sex was not independently associated with the overall VAX score. This pattern suggests that gender differences in vaccination attitudes may be more closely related to perceived safety uncertainty than to a broader anti-vaccination orientation. This pattern is consistent with international evidence indicating that gender differences in vaccination attitudes are contextual and heterogeneous: in some countries, female sex is associated with a lower likelihood of intending to be vaccinated [23], yet the magnitude and direction of gender effects vary depending on the type of vaccine, the study period, and the sociocultural setting [22]. Thus, gender differences appear to operate primarily at the level of specific cognitive components of vaccination attitudes, rather than necessarily being reflected in overall indicators of support for or rejection of vaccination.

Age demonstrated the most consistent directional pattern in the study. Compared with respondents aged 55 years and older, younger groups generally tended to have lower VAX scores, although the magnitude and statistical strength of these differences varied across domains. The clearest contrasts were observed in the youngest age groups, suggesting that older age in Kazakhstan may be associated with more sceptical vaccination attitudes. A similar tendency was reported by Tarasi et al. [24], in whose study age showed a statistically significant association with anti-vaccination attitudes: older participants were characterized by more negative attitudes toward vaccination, whereas younger individuals showed more positive views.

An important finding was the independent association with marital status: respondents who were married or cohabiting demonstrated lower scores across all VAX subscales and on the overall score compared with single participants. This may reflect the role of social support and family responsibility in shaping trust in preventive interventions and medical recommendations.

One of the most practically relevant findings was the association between diabetes and higher adjusted scores for mistrust, concerns about commercial profiteering, and the overall VAX score. This result is important because adults with diabetes represent a group for whom vaccine-preventable infections may carry higher clinical risk. At the same time, the present data do not allow identification of the mechanisms underlying this association, which may involve treatment burden, prior healthcare experiences, or disease-related perceptions of vulnerability. Overall, this pattern is consistent with the literature showing that even among people with chronic diseases who objectively belong to groups at elevated risk of infectious complications, vaccine hesitancy may remain substantial. In a systematic review and meta-analysis, Bianchi et al. [25] showed that approximately 27–28% of patients with diabetes demonstrate hesitancy, predominantly related to concerns about safety and effectiveness. At the same time, the review by Ekpor et al. [26] indicates a high pooled level of vaccine acceptance (approximately 76%) in this group, underscoring the possibility that high willingness to be vaccinated may coexist with pronounced cognitive doubts. Wang et al. [27] describe diabetes-specific concerns related to the potential effects of vaccination on disease control, whereas Kolobov et al. [28] report higher overall confidence in vaccine safety among patients with diabetes compared with the general population, despite persistent within-group variability in attitudes. Taken together, these findings suggest that the observed differences may be interpreted less as evidence of generalized fears about long-term vaccine effects than as reflecting stronger mistrust of vaccine benefit and greater sensitivity to issues of institutional and commercial integrity.

Territorial variation was present but not uniform across attitudinal domains. Urban residence was associated with lower mistrust and higher worries, while macro-region was significant at the factor level only for worries. This pattern suggests that geographical context may shape specific dimensions of vaccine attitudes rather than producing a single, generalisable territorial gradient. Such differences likely reflect not a territorial effect per se, but rather unequal distribution of information flows, access to health communication, institutional trust, and patterns of interaction with the healthcare system. This may be particularly relevant for urban–rural differences, where exposure to online information sources, access to health communication, and patterns of contact with healthcare services may differ substantially. The relatively low R^2^ values suggest that important determinants of vaccination attitudes remain unmeasured, particularly psychological, informational, and trust-related factors such as institutional trust, misinformation exposure, perceived risk, and prior vaccination experiences.

In studies conducted in China, with a combined sample of more than 6000 respondents, institutional trust showed a pronounced negative association with vaccine hesitancy, whereas perceived vaccine risk demonstrated a strong positive association [29,30]. A Canadian study involving 1541 adults found that individuals with high hesitancy were characterized by substantially lower levels of trust in government and medical institutions, while interpersonal trust showed no comparable relationship, suggesting that institutional trust may function as an independent protective factor [31].

The role of the information environment is also fundamental. An analysis conducted in France demonstrated that social media use combined with low institutional trust increases unwillingness to vaccinate, with both direct and indirect effects [32]. In the United Kingdom, obtaining information from unregulated online sources was associated with higher levels of hesitancy, whereas trust in medical experts and official sources reduced its expression [33]. Norwegian data further confirm that preference for uncontrolled media platforms, including forums, blogs, and social networks, is associated with stronger vaccine-related doubts [34].

In light of these findings, potential measures to reduce anti-vaccination attitudes in Kazakhstan should be multilevel and aligned with the factors found to be associated with the level of anti-vaccination attitudes.

Age-oriented communication appears necessary, as older groups demonstrated more pronounced negative attitudes; for these groups, priority should be given to clear explanations of safety issues and long-term observational evidence, delivered primarily through face-to-face formats via primary healthcare services and local communities. Regional heterogeneity requires adaptation of communication strategies to the local social and informational context, including the involvement of regional opinion leaders. Patients with diabetes require integration of vaccination discussions into the routine management of chronic disease, with emphasis on safety and clinical benefits for this risk group. A key condition for long-term impact remains the strengthening of institutional trust and the transition to a proactive communication model: transparency in decision-making, regular dissemination of safety data, and an active presence of official institutions in the digital environment may reduce uncertainty and contribute to a more sustained reduction in vaccine hesitancy. From a public health perspective, these findings support the value of targeted rather than uniform communication strategies. In particular, messages addressing long-term safety concerns and misconceptions about natural immunity may be especially important. Tailoring communication to older adults and to clinically vulnerable groups such as people with diabetes may be more effective than relying solely on broad population-level messaging.

## 5. Conclusions

This nationally representative study showed that anti-vaccination attitudes among adults in Kazakhstan are multidimensional and socially patterned. Safety-related concerns, particularly worries about unforeseen future effects, represent the most prominent attitudinal domain, while mistrust of vaccine benefit appears less pronounced. These findings indicate that vaccine hesitancy in Kazakhstan should be addressed through targeted communication strategies tailored to specific attitudinal domains and population subgroups.

## 6. Study Limitations

Several limitations should be considered when interpreting the findings of this study. First, the cross-sectional design precludes temporal inference. Although covariate selection was informed by a prespecified conceptual framework (DAG), the observed relationships represent adjusted associations rather than causal effects, and reverse causation or bidirectional relationships cannot be excluded.

Second, vaccination attitudes were assessed using a self-reported psychometric instrument. Although the VAX scale showed good overall reliability and acceptable structural validity in this sample, responses may still have been influenced by social desirability, response style, and context-dependent interpretation of items. Item V4 showed weaker item-level discrimination compared with the other items in its subscale, and its removal slightly increased Cronbach’s α. Nevertheless, the “worries about unforeseen future effects” subscale demonstrated acceptable internal consistency overall (α = 0.723), suggesting that this finding is more likely related to a single item than to poor performance of the subscale as a whole.

Third, despite adjustment for a broad range of socio-demographic, behavioural, and clinical variables, residual confounding remains possible. The survey did not directly measure several theoretically relevant psychosocial constructs, including institutional trust, exposure to misinformation, perceived risk, and prior vaccination experiences. These unmeasured contextual factors may have contributed to the limited explained variance of the regression models. In addition, separate models were fitted across five correlated outcomes, increasing the number of statistical comparisons, although interpretation focused primarily on effect sizes, confidence intervals, and consistency across related outcomes rather than isolated *p*-values.

Fourth, although the survey was nationally representative and design-corrected, certain territorial subgroup estimates and regression coefficients, particularly for sparsely populated areas, may be less stable because of smaller effective sample sizes. A similar caution applies to very small socio-demographic categories, particularly primary education, for which confidence intervals were relatively wide.

Finally, clinical characteristics were based on survey and measurement data collected at a single time point. The study did not capture disease severity, duration, treatment adherence, or other markers of within-group heterogeneity, which may be particularly relevant for diabetes, where disease duration, treatment type, glycaemic control, and complication burden could influence vaccination attitudes.

## Figures and Tables

**Table 1:** Socio-demographic, behavioural and clinical characteristics of the study population (n = 6712).

Characteristic	Category	n	% (95% CI)
Sex	Male	3196	47.6 (46.4–48.8)
	Female	3516	52.4 (51.2–53.6)
Age group (years)	18–24	911	13.6 (12.8–14.4)
	25–34	1433	21.3 (20.4–22.3)
	35–44	1636	24.4 (23.4–25.4)
	45–54	1226	18.3 (17.4–19.2)
	≥55	1506	22.4 (21.5–23.5)
Ethnicity	Turkic	5159	76.9 (75.8–77.9)
	Slavic	1191	17.7 (16.8–18.7)
	Other	362	5.4 (4.9–6.0)
Marital status	Married/cohabiting	4114	62.2 (61.0–63.4)
	Single	2499	37.8 (36.6–39.0)
	Missing	99	-
Education	Primary	28	0.4 (0.3–0.6)
	Secondary	2760	41.4 (40.2–42.5)
	Higher	3886	58.2 (57.0–59.4)
	Missing	38	-
Occupation	Public sector	1411	21.3 (20.3–22.3)
	Private sector	3514	53.0 (51.8–54.2)
	Students	275	4.1 (3.7–4.7)
	Homemakers	419	6.3 (5.8–6.9)
	Pensioners	640	9.7 (9.0–10.4)
	Unemployed	369	5.6 (5.0–6.1)
	Missing	84	-
Body mass index (BMI)	Underweight	210	3.2 (2.8–3.6)
	Normal weight	2483	37.5 (36.3–38.7)
	Overweight	2392	36.1 (35.0–37.3)
	Obesity	1538	23.2 (22.2–24.3)
	Missing	89	-
Blood pressure status	Normotensive	2738	40.8 (39.7–42.0)
	Pre-hypertensive	2251	33.6 (32.4–34.7)
	Hypertensive	1718	25.6 (24.6–26.7)
	Missing	5	-
Diabetes status	No diabetes	5958	88.8 (88.0–89.5)
	Diabetes	754	11.2 (10.5–12.0)
Current smoking	Yes	1552	23.3 (22.3–24.3)
	No	5110	76.7 (75.7–77.7)
	Missing	50	-
HED	No	6069	90.4 (89.7–91.1)
	Yes	643	9.6 (8.9–10.3)
Place of residence	Urban	4194	63.2 (62.0–64.3)
	Rural	2447	36.8 (35.7–38.0)
	Missing	71	-
Region of residence	North	1069	15.9 (15.1–16.8)
	Central	479	7.1 (6.5–7.8)
	East	465	6.9 (6.3–7.6)
	South	1990	29.6 (28.6–30.8)
	West	1031	15.4 (14.5–16.2)
	Astana city	502	7.5 (6.9–8.1)
	Almaty city	791	11.8 (11.0–12.6)
	Shymkent city	385	5.7 (5.2–6.3)

**Table 2:** Design-adjusted weighted mean scores and internal consistency of the VAX total scale and its subscales (n = 6536).

Scale/Subscale	Items	Mean (95% CI)	Cronbach’s α
VAX total score (1–6)	V1–V12	3.70 (3.67–3.73)	0.861
Mistrust of vaccine benefit	V1–V3	3.27 (3.23–3.31)	0.926
Worries about unforeseen future effects	V4–V6	4.12 (4.10–4.14)	0.723
Concerns about commercial profiteering	V7–V9	3.49 (3.45–3.52)	0.877
Preference for natural immunity	V10–V12	3.93 (3.87–3.98)	0.826

**Table 3:** Design-adjusted mean scores of vaccination attitude subscales across socio-demographic, behavioural, and clinical characteristics (weighted means and 95% CIs).

Category	Mistrust of Vaccine Benefit	Worries About Unforeseen Future Effects	Concerns About Commercial Profiteering	Preference for Natural Immunity
Sex				
Men	3.33 (3.19–3.46)	4.02 (3.93–4.11)	3.44 (3.34–3.55)	3.92 (3.80–4.03)
Women	3.24 (3.13–3.36)	4.15 (4.07–4.24)	3.50 (3.40–3.60)	3.92 (3.81–4.03)
Age group				
18–24	3.01 (2.84–3.19)	3.87 (3.75–4.00)	3.10 (2.98–3.22)	3.68 (3.54–3.81)
25–34	3.21 (3.06–3.37)	3.97 (3.87–4.07)	3.28 (3.15–3.40)	3.75 (3.62–3.88)
35–44	3.31 (3.17–3.45)	4.15 (4.05–4.25)	3.55 (3.43–3.66)	3.98 (3.85–4.11)
45–54	3.38 (3.25–3.51)	4.17 (4.08–4.26)	3.61 (3.48–3.74)	4.00 (3.88–4.12)
55+	3.41 (3.29–3.53)	4.20 (4.10–4.30)	3.70 (3.58–3.82)	4.10 (3.98–4.21)
Ethnicity				
Turkic	3.28 (3.15–3.40)	4.09 (4.00–4.18)	3.48 (3.38–3.57)	3.94 (3.83–4.04)
Slavic	3.29 (3.16–3.43)	4.12 (4.00–4.23)	3.43 (3.29–3.57)	3.81 (3.66–3.96)
Others	3.35 (3.12–3.57)	4.01 (3.85–4.18)	3.54 (3.28–3.80)	4.04 (3.84–4.25)
Education				
Primary education	4.47 (3.54–5.40)	2.98 (1.86–4.10)	2.67 (1.58–3.76)	2.98 (1.74–4.22)
Secondary education	3.37 (3.24–3.51)	4.09 (4.00–4.18)	3.50 (3.37–3.63)	3.94 (3.83–4.05)
Higher education	3.20 (3.09–3.31)	4.10 (4.01–4.19)	3.46 (3.37–3.56)	3.91 (3.80–4.03)
Marital status				
Married/ Cohabiting	3.27 (3.15–3.38)	4.09 (4.01–4.18)	3.50 (3.40–3.60)	3.94 (3.83–4.05)
Single	3.31 (3.18–3.45)	4.09 (3.99–4.19)	3.45 (3.34–3.57)	3.91 (3.80–4.02)
Occupation				
Employed in the public sector	3.08 (2.94–3.22)	4.11 (4.01–4.21)	3.48 (3.36–3.59)	3.88 (3.75–4.01)
Employed in the private sector	3.37 (3.24–3.49)	4.09 (4.00–4.18)	3.49 (3.39–3.59)	3.92 (3.82–4.03)
Students	2.82 (2.61–3.03)	3.81 (3.65–3.97)	2.94 (2.74–3.13)	3.51 (3.35–3.67)
Homemakers	3.34 (3.15–3.53)	4.12 (3.98–4.26)	3.50 (3.33–3.68)	3.97 (3.81–4.13)
Pensioners	3.40 (3.23–3.57)	4.24 (4.10–4.37)	3.65 (3.49–3.81)	4.13 (3.98–4.28)
Unemployed	3.31 (3.02–3.60)	4.02 (3.85–4.19)	3.47 (3.27–3.66)	4.08 (3.86–4.30)
BMI category				
Underweight (<18.5)	3.34 (3.07–3.62)	4.02 (3.81–4.23)	3.21 (2.98–3.45)	3.79 (3.59–3.99)
Normal weight (18.5–24.99)	3.23 (3.10–3.37)	4.04 (3.95–4.14)	3.40 (3.30–3.49)	3.86 (3.75–3.97)
Overweight (25.0–29.9)	3.32 (3.19–3.45)	4.12 (4.03–4.21)	3.53 (3.43–3.63)	3.95 (3.84–4.06)
Obesity (≥30.0)	3.30 (3.17–3.42)	4.11 (4.01–4.21)	3.53 (3.40–3.67)	3.97 (3.85–4.09)
Blood pressure status				
Normal BP	3.17 (3.04–3.29)	4.09 (3.99–4.18)	3.37 (3.26–3.48)	3.89 (3.78–4.00)
Pre-hypertension	3.32 (3.19–3.46)	4.02 (3.93–4.11)	3.47 (3.36–3.58)	3.89 (3.77–4.01)
Hypertension	3.41 (3.27–3.54)	4.18 (4.09–4.28)	3.63 (3.52–3.74)	4.00 (3.90–4.11)
Diabetes status				
No Diabetes	3.26 (3.14–3.37)	4.07 (3.98–4.16)	3.44 (3.34–3.54)	3.91 (3.80–4.02)
Diabetes	3.50 (3.37–3.64)	4.24 (4.13–4.34)	3.75 (3.63–3.87)	4.01 (3.90–4.12)
Smoking				
Yes	3.35 (3.20–3.50)	4.06 (3.96–4.16)	3.52 (3.38–3.65)	3.91 (3.78–4.04)
No	3.26 (3.15–3.38)	4.10 (4.02–4.18)	3.46 (3.37–3.55)	3.92 (3.82–4.03)
HED				
No HED	3.28 (3.16–3.40)	4.08 (4.00–4.17)	3.45 (3.35–3.55)	3.93 (3.82–4.03)
HED (≥1 time)	3.31 (3.19–3.43)	4.14 (4.04–4.24)	3.67 (3.54–3.81)	3.84 (3.69–3.99)
Place of residence				
Urban	3.17 (3.07–3.28)	4.23 (4.13–4.33)	3.50 (3.41–3.60)	3.90 (3.80–3.99)
Rural	3.44 (3.22–3.66)	3.89 (3.76–4.02)	3.43 (3.25–3.61)	3.95 (3.73–4.16)
Macro-region				
North	3.24 (3.15–3.33)	4.30 (4.24–4.36)	3.52 (3.44–3.60)	4.01 (3.94–4.08)
Central	3.22 (3.08–3.36)	3.87 (3.78–3.97)	3.20 (3.08–3.32)	3.79 (3.66–3.92)
East	3.27 (3.14–3.40)	4.30 (4.21–4.39)	3.81 (3.68–3.94)	4.08 (3.96–4.21)
South	3.24 (3.18–3.31)	3.87 (3.82–3.91)	3.41 (3.36–3.47)	3.93 (3.88–3.98)
West	3.68 (3.58–3.77)	4.15 (4.08–4.22)	3.60 (3.51–3.68)	3.86 (3.79–3.93)
Astana city	3.18 (3.06–3.31)	4.33 (4.24–4.41)	3.65 (3.54–3.75)	3.94 (3.84–4.04)
Almaty city	3.06 (2.95–3.16)	4.27 (4.19–4.35)	3.40 (3.30–3.50)	3.80 (3.71–3.89)
Shymkent city	3.05 (2.87–3.22)	4.35 (4.21–4.49)	3.46 (3.31–3.61)	4.07 (3.94–4.21)

Estimates for the primary education category should be interpreted cautiously because of the small number of respondents in this group.

## Data Availability

All available data was presented within manuscript.

## Supplementary Materials

The following supporting information can be downloaded at: https://www.mdpi.com/article/10.3390/vaccines14040353/s1, Figure S1: Conceptual causal diagram (directed acyclic graph, DAG) illustrating the assumed relationships between socio-demographic, behavioural, clinical characteristics and vaccination attitudes (VAX score); Table S1: Item-level descriptive statistics and reliability indices (n = 6536); Table S2: Design-adjusted multivariable linear regression analysis of factors associated with the Mistrust of Vaccine Benefit subscale (n = 6333); Table S3: Design-adjusted multivariable linear regression analysis of factors associated with the Worries about Unforeseen Future Effects subscale (n = 6332); Table S4: Design-adjusted multivariable linear regression analysis of factors associated with the Concerns about Commercial Profiteering subscale (n = 6333); Table S5: Design-adjusted multivariable linear regression analysis of factors associated with the Preference for Natural Immunity subscale (n = 6335); Table S6: Design-adjusted multivariable linear regression analysis of factors associated with the overall vaccination attitudes score (n = 6336).

## Abbreviations

The following abbreviations are used in this manuscript:

Abbreviation	Definition
BMI	Body mass index
BP	Blood pressure
CFA	Confirmatory factor analysis
CFI	Comparative fit index
CI	Confidence interval
COVID-19	Coronavirus disease 2019
DAG	Directed acyclic graph
DBP	Diastolic blood pressure
EDTA	Ethylenediaminetetraacetic acid
HbA1c	Glycated hemoglobin
HDL-C	High-density lipoprotein cholesterol
HED	Heavy episodic drinking
ITC	International Test Commission
LDL-C	Low-density lipoprotein cholesterol
PHC	Primary health care
PSU	Primary sampling unit
RMSEA	Root mean square error of approximation
SBP	Systolic blood pressure
SOPs	Standard operating procedures
STEPS	STEPwise approach to noncommunicable disease risk-factor surveillance
TLI	Tucker–Lewis index
VAX	Vaccination Attitudes Examination
VIF	Variance inflation factor
WHO	World Health Organization
