# Supplementary material for: Vaccination Attitudes in the Adult Population of Kazakhstan: A Nationally Representative Cross-Sectional Study
Source: Vaccines (Basel). 2026 Apr 16;14(4):353. doi: 10.3390/vaccines14040353 (PMC13119652; doi:10.3390/vaccines14040353)

Supplementary materials

Table S1. Item-level descriptive statistics and reliability indices (n = 6,536).

Item	**Short wording**	Mean±SD	r (item–total)	α if deleted	Missing, %
V1	I feel safe after being vaccinated	3.34±1.60	0.535	0.851	0.19
V2	I can rely on vaccines to prevent serious infectious diseases	3.18±1.57	0.516	0.852	0.21
V3	I feel protected after being vaccinated	3.29±1.57	0.525	0.851	0.37
V4	Although most vaccines appear to be safe, there may be problems that are not yet known about	4.05±1.33	0.162	0.872	0.18
V5	Vaccines can cause unforeseen problems in children	4.17±1.35	0.537	0.850	0.21
V6	I worry about unknown future effects of vaccines	4.13±1.41	0.568	0.848	0.45
V7	Vaccines make a lot of money for pharmaceutical companies, but do little for ordinary people	3.70±1.46	0.680	0.841	0.27
V8	Authorities promote vaccination for financial gain rather than for people's health	3.47±1.46	0.697	0.839	0.19
V9	Vaccination programmes are a big con	3.27±1.48	0.688	0.840	0.24
V10	Natural immunity lasts longer than immunity gained through vaccination	4.08±1.39	0.548	0.850	0.18
V11	Natural exposure to viruses and germs provides the safest protection	4.00±1.36	0.507	0.852	0.33
V12	Naturally acquired disease is safer for the immune system than vaccination	3.67±1.46	0.504	0.853	0.83

Table S2. Design-adjusted multivariable linear regression analysis of factors associated with the Mistrust of Vaccine Benefit subscale (n = 6333). Model fit: $R^2 = 0.046$; Wald $F(31,120) = 4.35$; $p < 0.001$.

**Variable**	**Category**	**β (95% CI)**	**p-value**	**Wald F (factor)**
**Sex**	Male	0.076 (−0.012; 0.164)	0.089	2.938
	Female	Ref.	—	
**Age group**	18–24	−0.394 (−0.603; −0.184)	<0.001	5.032
	25–34	−0.153 (−0.301; −0.006)	0.041	
	35–44	−0.024 (−0.168; 0.120)	0.74	
	45–54	0.028 (−0.104; 0.161)	0.67	
	55+	Ref.	—	
**Ethnicity**	Turkic	0.007 (−0.199; 0.213)	0.94	0.300

	Slavic	−0.041 (−0.244; 0.161)	0.69	
	Others	Ref.	—	
**Education**	Primary	0.785 (−0.067; 1.637)	0.072	3.008
	Secondary	0.083 (−0.014; 0.181)	0.095	
	Higher	Ref.	—	
**Marital status**	Married/Cohabiting	−0.187 (−0.277; −0.096)	<0.001	16.663
	Single	Ref.	—	
**Occupation**	Public sector	−0.240 (−0.473; −0.008)	0.043	6.305
	Private sector	0.031 (−0.203; 0.265)	0.79	
	Students	−0.318 (−0.631; −0.004)	0.047	
	Homemakers	0.085 (−0.203; 0.373)	0.56	
	Pensioners	−0.073 (−0.333; 0.186)	0.57	
	Unemployed	Ref.	—	
**BMI category**	Underweight	0.299 (0.026; 0.572)	0.032	1.584
	Normal	0.074 (−0.048; 0.195)	0.23	
	Overweight	0.050 (−0.072; 0.173)	0.42	
	Obesity	Ref.	—	
**Blood pressure status**	Normotensive	−0.125 (−0.278; 0.028)	0.11	1.378
	Pre-hypertensive	−0.040 (−0.152; 0.071)	0.47	
	Hypertensive	Ref.	—	
**Diabetes status**	No diabetes	−0.183 (−0.303; −0.063)	0.003	9.050
	Diabetes	Ref.	—	
**Smoking**	Yes	0.024 (−0.067; 0.116)	0.60	0.281
	No	Ref.	—	
**HED**	No HED	0.016 (−0.115; 0.147)	0.81	0.058
	HED	Ref.	—	
**Place of residence**	Urban	−0.255 (−0.469; −0.041)	0.020	5.542
	Rural	Ref.	—	
**Macro-region**	North	0.106 (−0.487; 0.699)	0.73	1.426
	Central	0.177 (−0.499; 0.853)	0.61	
	East	0.163 (−0.409; 0.736)	0.58	
	South	0.053 (−0.548; 0.654)	0.86	
	West	0.513 (−0.115; 1.142)	0.11	
	Astana city	0.171 (−0.418; 0.761)	0.57	
	Almaty city	−0.020 (−0.622; 0.583)	0.95	
	Shymkent city	Ref.	—	

Table S3. Design-adjusted multivariable linear regression analysis of factors associated with the Worries about Unforeseen Future Effects subscale (n = 6332). Model fit: $R^2 = 0.062$; Wald $F(31.120) = 3.97$; $p < 0.001$.

Variable	Category	β (95% CI)	p-value	Wald F (factor)
**Sex**	Men	−0.086 (−0.154; −0.018)	0.014	6.18
	Women	Ref.	—	
**Age group**	18–24	−0.284 (−0.412; −0.156)	<0.001	8.74
	25–34	−0.220 (−0.327; −0.112)	<0.001	
	35–44	−0.033 (−0.128; 0.063)	0.51	
	45–54	−0.012 (−0.093; 0.070)	0.78	
	55+	Ref.	—	
**Ethnicity**	Turkic	0.025 (−0.114; 0.164)	0.72	0.08
	Slavic	0.012 (−0.121; 0.144)	0.86	
	Others	Ref.	—	

**Education**	Primary	−0.544 (−1.505; 0.416)	0.26	1.97
	Secondary	0.039 (−0.021; 0.098)	0.20	
	Higher	Ref.	—	
**Marital status**	Married / cohabiting	−0.078 (−0.148; −0.008)	0.028	4.90
	Single	Ref.	—	
**Occupation**	Public sector	−0.168 (−0.473; 0.138)	0.28	0.34
	Private sector	−0.003 (−0.141; 0.135)	0.97	
	Students	−0.155 (−0.426; 0.117)	0.26	
	Homemakers	0.010 (−0.160; 0.180)	0.91	
	Pensioners	0.013 (−0.130; 0.156)	0.86	
	Unemployed	Ref.	—	
**BMI category**	Underweight	0.064 (−0.127; 0.256)	0.51	0.71
	Normal	0.002 (−0.075; 0.079)	0.96	
	Overweight	0.053 (−0.033; 0.139)	0.23	
	Obesity	Ref.	—	
**Blood pressure**	Normotensive	0.012 (−0.090; 0.114)	0.82	1.37
	Pre-hypertensive	−0.049 (−0.136; 0.038)	0.27	
	Hypertensive	Ref.	—	
**Diabetes**	No diabetes	−0.052 (−0.136; 0.032)	0.23	1.49
	Diabetes	Ref.	—	
**Smoking**	Yes	−0.007 (−0.079; 0.065)	0.84	0.04
	No	Ref.	—	
**HED**	No HED	−0.013 (−0.107; 0.081)	0.79	0.07
	HED	Ref.	—	
**Residence**	Urban	0.271 (0.125; 0.417)	<0.001	13.53
	Rural	Ref.	—	
**Macro-region**	North	0.106 (−0.487; 0.699)	0.73	2.79
	Central	−0.406 (−1.138; 0.326)	0.27	
	East	−0.008 (−0.696; 0.680)	0.98	
	South	−0.326 (−1.017; 0.365)	0.35	
	West	−0.122 (−0.811; 0.567)	0.73	
	Astana city	−0.038 (−0.743; 0.668)	0.91	
	Almaty city	−0.144 (−0.899; 0.611)	0.71	
	Shymkent city	Ref.	—	

Table S4. Design-adjusted multivariable linear regression analysis of factors associated with the Concerns about Commercial Profiteering subscale (n = 6333). Model fit: $R^2 = 0.046$; Wald $F(31,120) = 3.97$; $p < 0.001$.

Variable	Category	β (95% CI)	p-value	Wald F (factor)
**Sex**	Men	−0.043 (−0.123; 0.038)	0.30	1.20
	Women	Ref.	—	
**Age group**	18–24	−0.574 (−0.741; −0.406)	<0.001	9.40
	25–34	−0.428 (−0.554; −0.303)	<0.001	
	35–44	−0.143 (−0.268; −0.018)	0.025	
	45–54	−0.099 (−0.220; 0.022)	0.11	
	55+	Ref.	—	
**Ethnicity**	Turkic	−0.023 (−0.245; 0.199)	0.84	0.74
	Slavic	−0.118 (−0.320; 0.084)	0.25	
	Others	Ref.	—	
**Education**	Primary	−0.428 (−1.473; 0.618)	0.42	0.96
	Secondary	0.049 (−0.055; 0.152)	0.35	

	Higher	Ref.	—	
**Marital status**	Married / cohabiting	−0.139 (−0.235; −0.042)	0.005	7.99
	Single	Ref.	—	
**Occupation**	Public sector	−0.096 (−0.264; 0.072)	0.26	2.63
	Private sector	−0.047 (−0.217; 0.124)	0.59	
	Students	−0.342 (−0.604; −0.080)	0.011	
	Homemakers	0.002 (−0.221; 0.224)	0.99	
	Pensioners	−0.123 (−0.334; 0.089)	0.25	
	Unemployed	Ref.	—	
**BMI category**	Underweight	0.032 (−0.206; 0.270)	0.79	0.48
	Normal	0.044 (−0.063; 0.150)	0.42	
	Overweight	0.064 (−0.040; 0.168)	0.23	
	Obesity	Ref.	—	
**Blood pressure**	Normotensive	−0.070 (−0.188; 0.048)	0.24	0.89
	Pre-hypertensive	−0.008 (−0.097; 0.082)	0.86	
	Hypertensive	Ref.	—	
**Diabetes**	No diabetes	−0.165 (−0.269; −0.062)	0.002	9.90
	Diabetes	Ref.	—	
**Smoking**	Yes	0.035 (−0.063; 0.134)	0.48	0.50
	No	Ref.	—	
**HED**	No HED	−0.159 (−0.297; −0.020)	0.025	5.14
	HED	Ref.	—	
**Residence**	Urban	0.090 (−0.103; 0.283)	0.36	0.85
	Rural	Ref.	—	
**Macro-region**	North	0.037 (−0.323; 0.397)	0.84	0.96
	Central	−0.192 (−0.625; 0.242)	0.38	
	East	0.314 (−0.069; 0.696)	0.11	
	South	−0.004 (−0.344; 0.336)	0.98	
	West	0.154 (−0.217; 0.525)	0.41	
	Astana city	0.144 (−0.176; 0.465)	0.38	
	Almaty city	−0.128 (−0.509; 0.252)	0.51	
	Shymkent city	Ref.	—	

Table S5. Design-adjusted multivariable linear regression analysis of factors associated with the Preference for Natural Immunity subscale (n = 6335). Model fit: $R^2 = 0.033$; Wald $F(31,120) = 4.33$; $p < 0.001$.

Variable	Category	β (95% CI)	p-value	Wald F (factor)
**Sex**	Men	0.020 (−0.059; 0.098)	0.626	0.245
	Women	Ref.	—	
**Age group**	18–24	−0.455 (−0.596; −0.314)	<0.001	17.276
	25–34	−0.361 (−0.481; −0.240)	<0.001	
	35–44	−0.091 (−0.202; 0.021)	0.112	
	45–54	−0.081 (−0.187; 0.026)	0.138	
	55+	Ref.	—	

**Ethnicity**	Turkic	−0.077 (−0.234; 0.081)	0.338	5.891
	Slavic	−0.259 (−0.426; −0.091)	0.003	
	Others	Ref.	—	
**Education**	Primary	−0.579 (−1.812; 0.654)	0.357	0.773
	Secondary	0.033 (−0.062; 0.127)	0.495	
	Higher	Ref.	—	
**Marital status**	Married/Cohabiting	−0.102 (−0.185; −0.019)	0.016	5.889
	Single	Ref.	—	
**Occupation**	Public sector	−0.270 (−0.444; −0.095)	0.002	3.956
	Private sector	−0.183 (−0.344; −0.021)	0.027	
	Students	−0.444 (−0.697; −0.191)	0.001	
	Homemakers	−0.110 (−0.320; 0.100)	0.304	
	Pensioners	−0.131 (−0.313; 0.051)	0.157	
	Unemployed	Ref.	—	
**BMI category**	Underweight	0.013 (−0.173; 0.198)	0.892	0.113
	Normal	−0.020 (−0.109; 0.070)	0.666	
	Overweight	−0.002 (−0.084; 0.080)	0.960	
	Obesity	Ref.	—	
**Blood pressure**	Normotensive	0.038 (−0.061; 0.137)	0.453	0.363
	Pre-hypertensive	0.006 (−0.074; 0.085)	0.886	
	Hypertensive	Ref.	—	
**Diabetes**	No diabetes	0.011 (−0.102; 0.124)	0.851	0.036
	Diabetes	Ref.	—	
**Smoking**	Yes	−0.008 (−0.090; 0.074)	0.844	0.039
	No	Ref.	—	
**HED**	No HED	0.087 (−0.062; 0.236)	0.249	1.340
	HED	Ref.	—	
**Residence**	Urban	−0.054 (−0.274; 0.167)	0.633	0.228
	Rural	Ref.	—	
**Macro-region**	North	−0.105 (−0.858; 0.648)	0.784	0.947
	Central	−0.212 (−1.051; 0.627)	0.621	
	East	−0.029 (−0.889; 0.832)	0.948	
	South	−0.214 (−0.966; 0.538)	0.577	

	West	−0.251 (−1.004; 0.503)	0.515	
	Astana city	−0.155 (−0.892; 0.581)	0.679	
	Almaty city	−0.310 (−1.048; 0.428)	0.410	
	Shymkent city	Ref.	—	

Table S6. Design-adjusted multivariable linear regression analysis of factors associated with the overall vaccination attitudes score (n = 6336). Model fit: $R^2 = 0.052$; Wald $F(31,120) = 7.34$; $p < 0.001$.

Variable	Category	β (95% CI)	p-value	Wald F (factor)
**Sex**	Men	−0.009 (−0.061; 0.044)	0.751	0.101
	Women	Ref.	—	
**Age group**	18–24	−0.427 (−0.532; −0.321)	<0.001	21.712
	25–34	−0.289 (−0.379; −0.199)	<0.001	
	35–44	−0.072 (−0.154; 0.011)	0.088	
	45–54	−0.041 (−0.118; 0.037)	0.304	
	55+	Ref.	—	
**Ethnicity**	Turkic	−0.017 (−0.150; 0.117)	0.806	3.186
	Slavic	−0.101 (−0.224; 0.022)	0.108	
	Others	Ref.	—	
**Education**	Primary	−0.190 (−0.841; 0.460)	0.565	1.856
	Secondary	0.051 (−0.007; 0.110)	0.085	
	Higher	Ref.	—	
**Marital status**	Married/Cohabiting	−0.126 (−0.190; −0.062)	<0.001	15.301
	Single	Ref.	—	
**Occupation**	Public sector	−0.155 (−0.268; −0.042)	0.007	5.960
	Private sector	−0.051 (−0.164; 0.063)	0.378	
	Students	−0.314 (−0.500; −0.129)	0.001	
	Homemakers	−0.004 (−0.166; 0.158)	0.962	
	Pensioners	−0.079 (−0.218; 0.061)	0.268	
	Unemployed	Ref.	—	
**BMI category**	Underweight	0.102 (−0.065; 0.269)	0.230	0.885
	Normal	0.025 (−0.045; 0.095)	0.486	
	Overweight	0.042 (−0.029; 0.113)	0.243	
	Obesity	Ref.	—	
**Blood pressure**	Normotensive	−0.037 (−0.119; 0.046)	0.380	0.395
	Pre-hypertensive	−0.023 (−0.085; 0.040)	0.473	
	Hypertensive	Ref.	—	
**Diabetes**	No diabetes	−0.099 (−0.171; −0.027)	0.008	7.297
	Diabetes	Ref.	—	
**Smoking**	Yes	0.010 (−0.050; 0.071)	0.729	0.121
	No	Ref.	—	
**HED**	No HED	−0.017 (−0.106; 0.072)	0.700	0.149
	HED	Ref.	—	
**Residence**	Urban	0.013 (−0.108; 0.135)	0.827	0.048

	Rural	Ref.	—	
**Macro-region**	North	0.014 (−0.306; 0.334)	0.932	1.448
	Central	−0.158 (−0.584; 0.267)	0.462	
	East	0.109 (−0.239; 0.458)	0.535	
	South	−0.123 (−0.450; 0.205)	0.460	
	West	0.074 (−0.260; 0.408)	0.662	
	Astana city	0.031 (−0.280; 0.341)	0.844	
	Almaty city	−0.151 (−0.516; 0.215)	0.416	
	Shymkent city	Ref.	—	

Figure S1. Conceptual causal diagram (directed acyclic graph, DAG) illustrating the assumed relationships between socio-demographic, behavioural, clinical characteristics and vaccination attitudes (VAX score).

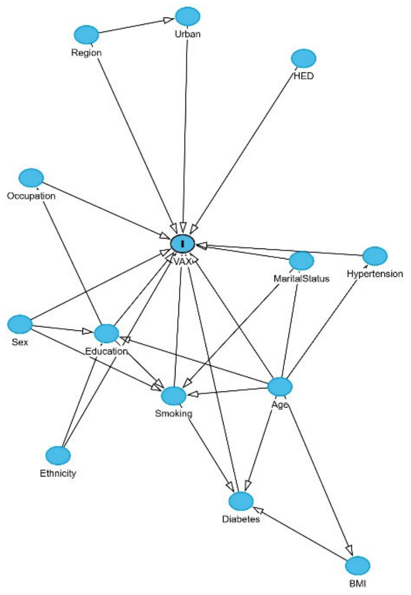

Supplement: Supplementary file 1 [file vaccines-14-00353-s001.zip › vaccines-4234730-supplementary.pdf]
